# Molecular Characterization of the Bacterial Community in a Potato Phytosphere

**DOI:** 10.1264/jsme2.ME13006

**Published:** 2013-06-08

**Authors:** Nobutaka Someya, Yuki Ohdaira Kobayashi, Shogo Tsuda, Seishi Ikeda

**Affiliations:** 1 Hokkaido Agricultural Research Center, National Agriculture and Food Research Organization, 9–4 Shinsei-minami, Memuro-cho, Kasai-gun, Hokkaido 082–0081, Japan; 2 Kyushu Okinawa Agricultural Research Center, National Agriculture and Food Research Organization, 6651–2 Yokoichi, Miyakonojo, Miyazaki 885–0091, Japan

**Keywords:** bacterial community, potato, phytosphere

## Abstract

The bacterial community of a potato phytosphere at the flowering stage was examined using both culture-dependent and -independent methods. Tissues (leaves, stems, roots and tubers) were sampled from field-grown potato plants (cultivar Matilda), and the clone libraries of 16S rRNA genes and the isolate collections using R2A medium were constructed. By analyzing the combined data set of 16S rRNA gene sequences from both clone libraries and isolate collections, 82 genera from 8 phyla were found and 237 OTUs (≥97% identity) at species level were identified across the potato phytosphere. The statistical analyses of clone libraries suggested that stems harbor the lowest diversity among the tissues examined. The phylogenetic analyses revealed that the most dominant phylum was shown to be *Proteobacteria* for all tissues (62.0%–89.7% and 57.7%–72.9%, respectively), followed by *Actinobacteria* (5.0%–10.7% and 14.6%–39.4%, respectively). The results of principal coordinates analyses of both clone libraries and isolate collections indicated that distinct differences were observed between above- and below-ground tissues for bacterial community structures. The results also revealed that leaves harbored highly similar community structures to stems, while the tuber community was shown to be distinctly different from the stem and root communities.

A phytosphere is an attractive habitat for microbes due to the high availability of nutrients and the relatively stable environment under field conditions. The plant-associated microbes are considered to be one of the important environmental factors for plants as it is well known that these microbes can assist plants for the uptake of nutrients from soils and the suppression of pathogen infections. To date, numerous studies for surveying and characterizing beneficial plant-associated microbes have been conducted worldwide over a few decades (26, 31, 38, 51, 68); however, only limited success has been achieved for the development of commercial microbial products for the biological control and growth promotion of plants. Several factors account for the difficulty of commercial utilization of beneficial microbes. Among them, the inconsistency of product performances under field conditions is the most important technical issue in the utilization of beneficial microbes in an agronomic environment.

Under field conditions, the persistency and functions are still not well characterized for most plant-associated microbes (11, 52). Therefore, as pointed out by several research groups (4, 37), the successful utilization of beneficial microbes in agronomic environments largely depends on the comprehensive knowledge of plant–microbe interactions at a community level under field conditions. Thus, better understanding of the diversity and functionality of a plant-associated microbial community under field conditions would promote the utilization of beneficial microbes to increase plant growth and the biological control of plant pathogens in agricultural practices (4).

Potato (*Solanum tuberosum* L.) is one of the world’s most important crops. Since it was found that environmental microbes have intimate interactions with potato plants (17), phylogenetic and functional diversities of potato-associated microbes have been investigated, mainly by using culture-dependent methods (4, 7, 52, 64). These culture-dependent analyses revealed some degree of information about the phylogenetic and functional diversities of potato-associated microbes and identified several beneficial and deleterious microbes. However, it is now evident that community analyses by culture-dependent methods are seriously biased due to the lack of information about the growth requirements for most microbes in the environment and the status of cells that are known as viable but not culturable even for known culturable microbes (60). Moreover, another considerable bias in these previous studies was the intentional selection of different colony morphologies, which was aimed to gain more diversity than random selection. This causes a serious bias for species abundance in an ecological evaluation; therefore, an appropriate ecological assessment could not be conducted in most previous studies of plant-associated bacteria. In recent years, methodological advances have been made in the field of molecular microbial ecology by developing a series of sophisticated molecular tools. These advances can provide a less biased, more comprehensive picture of the diversity of environmental microbes without culturing environmental microbes, and could enhance the efficiency of the survey of beneficial microbes in a phytosphere. More importantly, they would allow assessments of the dynamics and functionality of a microbial community in a phytosphere in a practical agronomic environment.

Recently, a series of studies have reported the characteristics of the community structures of potato-associated bacteria analyzed by culture-independent methods (4, 14, 29, 44, 49, 50). These culture independent analyses revealed the tissue-specific distribution of potato-associated bacteria (29), and also showed that abiotic as well as biotic environmental factors have considerable impacts on the community structures of potato-associated bacteria (44, 50). More recently, massive sequencing technologies also have been employed for community analysis of the potato rhizosphere (25, 34). Although these culture-independent analyses have provided significant information to reveal the community structure in potato plants (14, 29, 45, 49), most of these studies have only focused on a rhizosphere- or tuber-associated community. Thus, a comprehensive investigation of microbial community structures has not been conducted for an entire phytosphere of potato plants, including both upper and under underground tissues.

Despite the successful application of diverse culture-independent methods to the analyses of microbial communities in a wide range of natural habitats, there is a serious limitation of these methodologies for analyzing the microbial community in a phytosphere due to a plant-inherent problem, which is the presence of an excess amount of plant DNA in the tissues. This causes outcompeting of plant DNA in the PCR amplification of 16S or 18S rRNA genes and considerably reduces the efficiency of sequencing derived from microbial DNAs, even with massive sequencing technologies. Hence, most culture-independent analyses of plant-associated bacteria have been limited to rhizosphere soil where the microbial biomass is relatively abundant in a phytosphere (49, 50).

In 2009, a method was developed for enriching bacterial cells from plant tissues (20). This cell enrichment method enables the comprehensive assessment of plant-associated bacteria in both above- and below-ground tissues by culture-independent analyses. In addition, recent advances in sequencing technologies and bioinformatics, a sequence-based community analysis, have provided powerful tools for obtaining unambiguous ecological information, considering both species richness and abundance. In conjunction with the cell enrichment method, such ecological assessments are now capable of providing data on plant-associated microbial communities for conducting efficient screening of beneficial microbes for reliable utilization under field conditions (21).

In the present study, the community structures of potato-associated bacteria in an entire phytosphere were examined at the flowering stage using both culture-dependent and -independent methods. The flowering stage was chosen and investigated using community analyses in the present study, since vegetative growth until the flowering stage is the main determinant for the entire productivity of potatoes. The results suggested the presence of tissue specificity for different taxonomical units ranging from phylum to species levels. This ecological information, such as the specificity and abundance in various tissues, obtained in the present study would be useful for surveying beneficial bacteria from a bacterial isolate collection for plant growth promotion and disease control in agricultural practices.

## Materials and Methods

### Plant materials and sampling

The cultivar “Matilda” was used for assessing the diversity of potato-associated bacteria. The seed tubers were planted on 27 April 2010 in an experimental field (42°89.2′ N/143°07.7′ E) at Memuro Research Station of Hokkaido Agricultural Research Center (Memuro, Hokkaido, Japan). The field was dressed with a commercial fertilizer (60, 170, and 102 kg for N, P, K ha^−1^) for basal fertilization. Plants at flowering time were sampled on 5 July 2010 and separated into leaves, stems, roots and tubers. Each tissue was washed with tap water and stored at −30°C until used for DNA extraction. Nine plants were sampled, and the individual plant was processed for bacterial cell enrichment, DNA extraction and PCR. General soil characteristics at the time of sampling were analyzed by Tokachi Nokyoren Agricultural Research Institute (Obihiro, Japan). Characteristics of the soil sample were as follows: soil type, andosol; pH 5.8; available P (Truog-P), 0.07 mg g^−1^; phosphate absorption coefficient, 1,591; cation exchange capacity, 0.18 me g^−1^; total nitrogen, 0.28%; available nitrogen, 46.1 g kg^−1^; humic content, 5.45%; CaO content, 0.31 mg g^−1^; MgO content, 0.31 mg g^−1^; K_2_O content, 0.15 mg g^−1^; NO_3_-N content, 17.1 g kg^−1^; and NH_4_-N content, 7.6 g kg^−1^.

### Isolation of potato-associated bacteria

Three potato plants at flowering time were sampled on 5 July 2010 and were immediately transported on ice to a laboratory. The plants were separated into leaves, stems, roots and tubers. Stems and tubers were washed well with tap water to remove loosely attached soil. Each tissue of three plants was combined and homogenized with phosphate buffer using a mortar and pestle. An aliquot of the homogenate was serially diluted and 100 μL aliquot from each dilution was spread onto a R2A (Difco, Detroit, MI, USA) agar plate containing cycloheximide at 25 μg mL^−1^. After incubation of the inoculated plates at 25°C for 7 d, bacterial colonies were detected at 8.8×10^7^ cfu g^−1^, 1.6×10^7^ cfu g^−1^, 6.2×10^7^ cfu g^−1^, and ca. 5.4×10^6^ cfu g^−1^ for leaves, stems, roots and tubers, respectively. Approximately 200 colonies were randomly picked up for each tissue. The bacteria were purified by single colony isolation, and genomic DNA was prepared as described previously (39).

### Clone library construction and sequencing

For each plant, approximately 50 g leaves or 100 g stems were homogenized with a buffer in a blender without surface sterilization to prepare leaf- and stem-associated bacterial cells (including both epiphytes and endophytes), and the cells were extracted and purified by an cell enrichment method (20). Approximately 20 g roots or 50 g tubers derived from an individual plant were ground into powder in liquid nitrogen with a mortar and pestle, and were used for cell extraction. Total DNA was extracted from an enriched bacterial cell sample by a DNA extraction method (23). A final DNA sample derived from an individual plant was suspended in 50 μL sterilized water. The quality and quantity of DNA were assessed spectrophotometrically by calculating absorbance at a wavelength of 260 nm (*A*_260_) and the *A*_260_/*A*_230_ and *A*_260_/*A*_280_ ratios. PCR clone libraries for 16S rRNA genes were constructed as follows. Briefly, 25 ng total bacterial DNA was used as a template in a final reaction volume of 12.5 μL, including 25 pmol of each primer and 1 U Ex *Taq* DNA polymerase (Takara Bio, Otsu, Japan). The universal primers 27F (5′-AGAGTTTGATCMTGGCTCAG-3′) and 1525R (5′-AAGGAGGTGWTCCARCC-3′) were used (30). Cycling conditions were as follows: initial denaturation for 2 min at 94°C; then 25 cycles consisting of 30 s at 94°C, 30 s at 55 °C, and 2 min at 72°C; and a final extension for 10 min at 72°C. PCR products derived from the same tissues of nine plants were combined into a composite sample, and the PCR product was resolved by 1% agarose gel electrophoresis in 1×TBE (89 mM Tris-Borate, 0.2 mM EDTA) buffer. The PCR product of predicted size (approximately 1,500 bp) was extracted from a gel using NucleoSpin Extract II (Macherey-Nagel, Düren, Germany) and was ligated into a pGEM-T Easy plasmid vector (Promega Japan, Tokyo, Japan) at 25°C for 1 h. Clone library construction and sequencing of 16S rRNA genes were carried by the Takara Bio Dragon Genomic Center (Takara Bio, Yokkaichi, Japan). A partial sequence of the 16S rRNA gene was obtained using the 27F primer. The 16S rRNA genes were amplified using a template DNA derived from isolate DNAs under the same PCR conditions as described for the construction of clone libraries, and direct sequencing was conducted by the Takara Bio Dragon Genomic Center (Takara Bio) using the 27F primer. Sequences were manually edited to eliminate primer sequences and low-quality regions. Approximately 500 bases of the 16S rRNA gene (corresponding to 109 to 665 bases of the *Escherichia coli* 16S rRNA gene) were then used for sequence analyses.

### Sequence analysis

Sequences were analyzed for orientation and detection of non-16S rRNA gene sequences using OrientationChecker (2). The presence of chimeras was assessed by MALLARD (2). A sequence identified at the 99.9% threshold was discarded as a chimera. The remaining sequences were aligned using CLUSTAL W (61). On the basis of the alignment, a distance matrix was constructed using the DNADIST program from PHYLIP ver. 3.66 (http://evolution.genetics.washington.edu/phylip.html) with the default parameters. The resulting matrices were run in Mothur (46) to generate diversity indexes and clustering analyses. The operational taxonomic units (OTUs) were defined with ≥97% identity for clustering analyses. Library coverage was calculated with the non-parametric estimator *C* (15), as described by Kemp and Aller (27). The reciprocal of Simpson’s index (1/*D*) was used as a measure of diversity to evaluate the level of dominance in a community (69). UniFrac (32) was applied to examine the similarities between clone libraries or isolate collections. A tree file generated by CLUSTAL W and an environment file, which links a file to a library, were uploaded to UniFrac. Principal coordinates analysis (PCoA) was performed by using UniFrac with the abundance-weighted option.

### Phylogenetic analysis

The phylogenetic composition of each clone library or isolate collection was evaluated by using the LibCompare program of RDP-II release 10 (65), with confidence levels of 80%. BLASTN (1) was also used to classify the clones and to identify the closest relatives in the public databases. For phylogenetic tree analyses, sequences were aligned using the CLUSTAL W program. The neighbor-joining method was used to build the trees (45). The PHYLIP format tree output was obtained by using the bootstrapping procedure (12); 1,000 bootstrap trials were used. The trees were constructed using TreeView software (40).

### Nucleotide sequence accession numbers

The nucleotide sequences reported in the present study were deposited in the DDBJ/EMBL/GenBank database. The sequence data of clone libraries for leaf, stem, root and tuber were deposited under accession numbers AB729140–AB729289, AB729290–AB729458, AB729459–AB729632 and AB729633–AB729793, respectively. The sequence data of isolate collections for leaf, stem, root and tuber were deposited under accession numbers AB729794–AB729998, AB729999–AB730173, AB730174–AB730371 and AB730372–AB730583, respectively.

## Results

### Statistical analyses of clone libraries and isolate collections

In the present study, the clone libraries and isolate collections were constructed for potato-associated bacteria for leaves, stems, roots and tubers. The statistical characteristics of these clone libraries and isolate collections are summarized in Table 1. The numbers of OTUs and diversity indexes for the libraries of leaf, stem, and root were clearly higher than those for the corresponding isolate collections as expected; however, in the case of tubers, the number of OTUs and diversity indexes for the isolate collections were shown to be higher than those for the clone library. The library coverage was considered to be experimentally high enough for most of the clone libraries and isolate collections (ranging from 83.9% to 98.5%), except the root clone library showing only 55.6% of library coverage. In both clone libraries and isolate collections, the highest diversity was observed in root-associated bacteria. Meanwhile, the stem- and leaf-associated bacteria were shown to have the lowest diversities in the clone libraries and isolate collections, respectively. By analyzing the combined data set from the clone libraries and isolate collections for all tissues, 82 genera from 8 phyla were found and 237 OTUs (clustering with ≥97% identity) were identified across the entire potato phytosphere.

### Phylogenetic analyses

The analyses of phylogenetic compositions by the LibCompare of RDP II revealed that the clone libraries were mainly dominated by 2 to 4 phyla (Table 2). The stem clone library consisted of only 2 phyla (*Proteobaceria* and *Actinobacteria*). The root clone library was shown to be the most diverse, containing 4 major phyla (*Proteobacteria*, *Actinobacteria*, *Frimicutes*, and *Planctomycetes*). The most dominant phylum among all libraries was *Proteobacteria*. In particular, leaf and stem clone libraries were shown to be highly dominated by *Proteobacteria* (84.0% and 89.7%, respectively). Similarly, the isolate collections were mainly dominated by only 2 or 3 phyla. *Proteobacteria* and *Actinobacteria* were the dominant phyla in the isolate collections for all tissues (57.7% to 72.9% and 14.7% to 39.4%, respectively), while *Bacteroidetes* was mainly observed in the isolate collections for below-ground tissues (12.1% and 21.7% for roots and tubers, respectively).

**Fig. 1:**
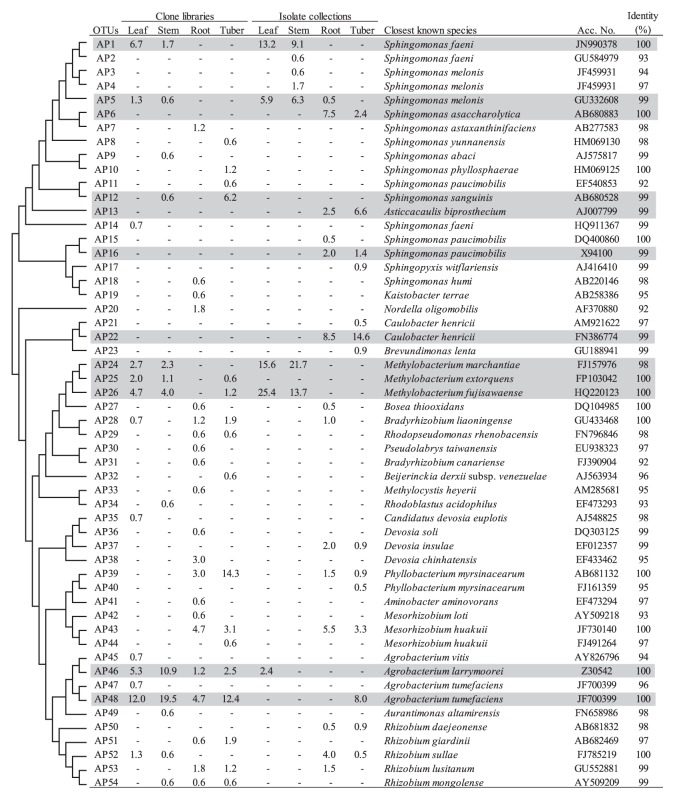
Phylogenetic distribution of OTUs for *Alphaproteobacteria* based on the 16S rRNA gene sequences of the clone libraries and isolate collections derived from field grown potato plants. The dendrogram indicates the phylogenetic relationships among the representative sequences of OTUs (defined by ≥97% identity). The table indicates the relative abundance of clones or isolates belonging to each OTU in each library or collection and the results of a BLAST search using the representative sequences. Shading indicates OTUs described in the main text.

Among the *Proteobacteria*, *Alphaproteobacteria* was the most dominant and was stably found in all clone libraries and isolate collections (Table 2). Most *Alphaproteobacteria* belonged to two orders *Rhizobiales* and *Sphingomonadales*. Within the order *Rhizobiales*, the group of *Rhizobium/Agrobacterium* was shown to be stably present in all clone libraries (8.9%–29.9%). Clustering analyses identified 2 OTUs (AP46 and AP48), which were distributed in all clone libraries (Fig. 1). The representative sequences of these OTUs were identical to *Agrobacterium larrymoorei* and *Agrobacterium tumefaciens* (Fig. 1). In contrast to the clone libraries, the group of *Rhizobium/Agrobacterium* in the isolate collections was mainly detected in the below-ground tissues (Table 2). The genus *Methylobacterium* was also found to be one of the predominant taxa in the clone libraries for above-ground tissues (9.3% and 7.5% for leaf and stem clone libraries, respectively) (Table 2), and the corresponding OTUs (AP24, AP25, and AP26) were identified (Fig. 1). Similarly, isolates of *Methylobacterium* sp. corresponding to OTUs AP24 and AP26 were obtained from above-ground tissues (41.0% and 35.4% for leaf and stem isolate collections, respectively) (Fig. 1). In contrast to *Methylobacterium* sp., two genera in the family *Phyllobacteriaceae* (*Mesorhizobium* and *Phyllobacterium*) were detected for only below-ground tissues in both clone libraries and isolate collections (Fig. 1). The genus *Caulobacter* was observed only in the isolate collections for the below-ground tissues (8.5% and 14.6% for root and tuber, respectively) (Table 2). All isolates of *Caulobacter* sp. belonged to OTU AP22 (Fig. 1).

In the order *Sphingomonadales*, the genus *Sphingomonas* was found to be present in both clone libraries and isolate collections for all tissues (Table 2); however, no OTU distribution across all tissues was identified for this genus by clustering analyses at species level (Fig. 1). Thus, OTU AP1 and OTU AP5 were exclusively detected in above-ground tissues for both clone libraries and isolate collections (Fig. 1). In contrast, OTU AP12 was shown to have relatively high abundance in the tuber clone library (6.2%) (Fig. 1). In addition, isolates belonging to 3 OTUs (AP6, AP13 and AP16) showed biased distribution to the below-ground tissues (Fig. 1).

**Fig. 2:**
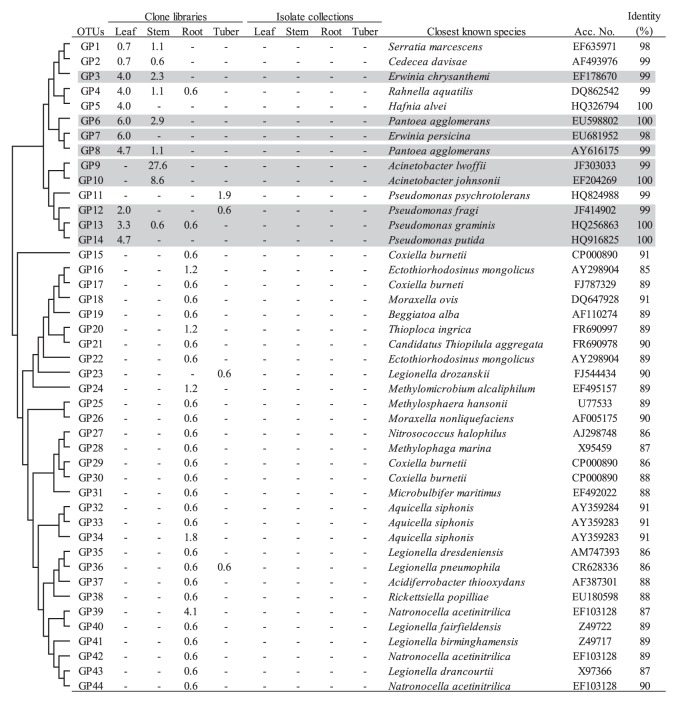
Phylogenetic distribution of OTUs for *Gammaproteobacteria* based on the 16S rRNA gene sequences of the clone libraries and isolate collections derived from field-grown potato plants. The dendrogram indicates the phylogenetic relationships among the representative sequences of OTUs (defined by ≥97% identity). The table indicates the relative abundance of clones or isolates belonging to each OTU in each library or collection and the results of a BLAST search using the representative sequences. Shading indicates OTUs described in the main text.

The *Gammaproteobacteria* was also found to be a dominant taxon in three libraries (leaf, stem and root libraries) with high abundance comparable to *Alphaproteobacteria*. Three genera (*Pantoea*, *Erwinia* and *Pseudomonas*, ranging from 4.7% to 10.0%) were responsible for the dominance of *Gammaproteobacteria* in the leaf clone library, while the genus *Acinetobacter* was exclusively found in the stem clone library (36.2%) (Table 2), and the corresponding two OTUs (GP9 and GP10) were identified (Fig. 2). The representative sequences of these OTUs showed 99% and 100% identity to *Acinetobacter lwoffii* and *Acinetobacter johnsonii*, respectively (Fig. 2). In analyses with the Classifier of RDPII, the high abundance of *Gammaproteobacteria* with uncertain phylogenetic affiliation was found in the root clone library (Table 2). This was also reflected in clustering analyses by the presence of a large cluster that is distantly related to known species of *Gammaproteobacteria* (OTUs corresponding to GP15 to GP44 in Fig. 2).

**Fig. 3:**
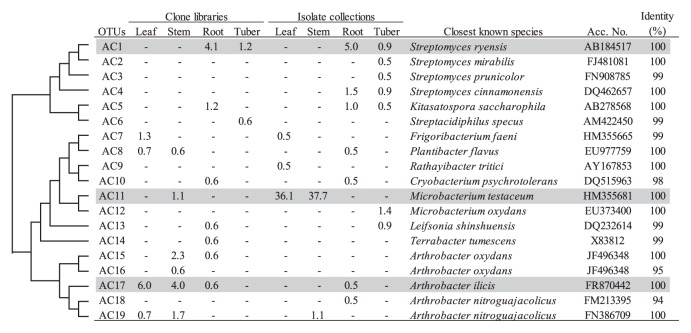
Phylogenetic distribution of OTUs for *Actinobacteria* based on the 16S rRNA gene sequences of the clone libraries and isolate collections derived from field-grown potato plants. The dendrogram indicates the phylogenetic relationships among the representative sequences of OTUs (defined by ≥97% identity). The table indicates the relative abundance of clones or isolates belonging to each OTU in each library or collection and the results of a BLAST search using the representative sequences. Shading indicates OTUs described in the main text.

In the *Actinobacteria*, the genus *Arthrobacter* was mainly found in the clone libraries for above-ground tissues (6.7% and 8.0% for leaf and stem, respectively) (Table 2), and the corresponding OTU (AC17) was identified (Fig. 3). In contrast, the genus *Streptomyces* was detected for only below-ground tissues in both clone libraries and isolate collections. One of the major differences in the phylogenetic compositions between the clone libraries and isolate collections was the extremely high abundance of the genus *Microbacterium* in the isolate collections for above-ground tissues (Table 2). All isolates were shown to belong to one OTU (AC11), and the representative sequence of this OTU was identical to *Microbacterium testaceum* (Fig. 3).

**Fig. 4:**
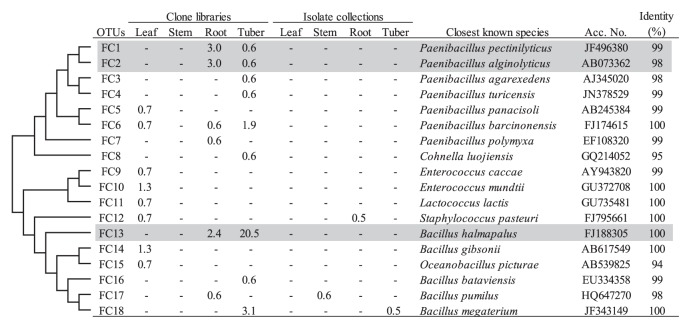
Phylogenetic distribution of OTUs for *Firmicutes* based on the 16S rRNA gene sequences of the clone libraries and isolate collections derived from field-grown potato plants. The dendrogram indicates the phylogenetic relationships among the representative sequences of OTUs (defined by ≥97% identity). The table indicates the relative abundance of clones or isolates belonging to each OTU in each library or collection and the results of a BLAST search using the representative sequences. Shading indicates OTUs described in the main text.

In the *Firmicutes*, the genus *Paenibacillus* was detected in the clone libraries for only below-ground tissues (7.1% and 5.0% for roots and tubers, respectively) (Table 2). While the tuber clones of *Paenibacillus* sp. belonged to several different OTUs showing the scattered phylogenetic distribution, most of the root clones for this genus belonged to two OTUs (FC1 and FC2) (Fig. 4). The genus *Bacillus* was shown to be extremely highly abundant in the tuber clone library (25.0%) (Table 2), and most tuber clones for this genus belonged to one OTU (FC13) (Fig. 4). The representative sequence of this OTU was identical to *Bacillus halmapalus*.

In the present study, *Planctomycetes* was only detected in the root clone library (8.9%) (Table 2). The genus *Schlesneria* was the most dominant in this phylum (4.1%) (Table 2 and Supplemental Fig. S1).

**Fig. 5:**
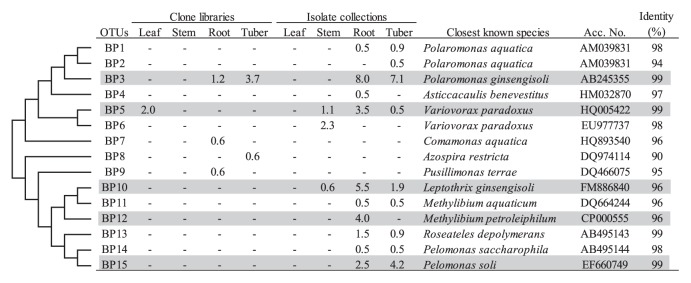
Phylogenetic distribution of OTUs for *Betaproteobacteria* based on the 16S rRNA gene sequences of the clone libraries and isolate collections derived from field-grown potato plants. The dendrogram indicates the phylogenetic relationships among the representative sequences of OTUs (defined by ≥97% identity). The table indicates the relative abundance of clones or isolates belonging to each OTU in each library or collection and the results of a BLAST search using the representative sequences. Shading indicates OTUs described in the main text.

One of other major differences in phylogenetic compositions between the clone libraries and isolate collections was the high abundance and high diversity of *Betaproteobacteria*, especially *Burkholderiales* bacteria, in the isolate collections for below-ground tissues (31.7% and 17.5% for roots and tubers, respectively) compared to those in the clone libraries. Within the *Burkholderiales*, three genera in the family *Comamonadaceae* (*Polaromonas*, *Variovorax* and *Pelomonas*) were found to be the dominant taxa (Table 2). Three OTUs corresponding to each of these genera were identified (BP3, BP5 and BP15) (Fig. 5). As another genus in the order *Burkholderiales*, the genus *Methylibium* was detected in the root isolate collection (4.5% in Table 2), and most isolates of *Methylibium* sp. belonged to OTU BP12 (Fig. 5). In addition, clustering analysis revealed that OTU BP10, closely related to *Leptothrix* sp., was also responsible for the high abundance of *Betaproteobacteria* in the root isolate collection (Fig. 5).

**Fig. 6:**
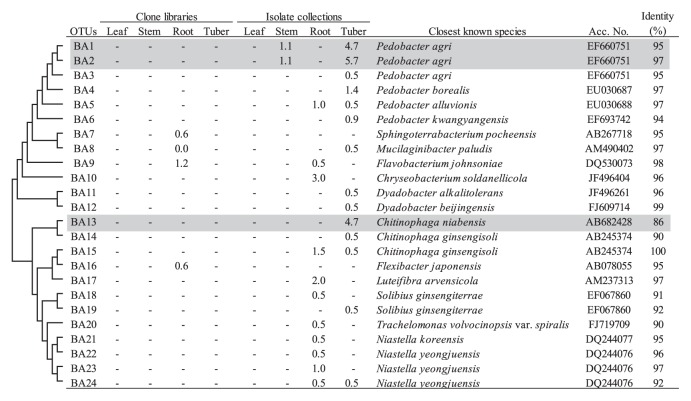
Phylogenetic distribution of OTUs for *Bacteroidetes* based on the 16S rRNA gene sequences of the clone libraries and isolate collections derived from field-grown potato plants. The dendrogram indicates the phylogenetic relationships among the representative sequences of OTUs (defined by ≥97% identity). The table indicates the relative abundance of clones or isolates belonging to each OTU in each library or collection and the results of a BLAST search using the representative sequences. Shading indicates OTUs described in the main text.

Similar to the *Betaproteobacteria*, high abundance of *Bacteroidetes* was found in the collections for below-ground tissues (12.1% and 21.7% for root and tuber, respectively) (Table 2). The abundance of *Pedobacter* sp. was especially high in the tuber collection (12.3% in Table 2), and the corresponding dominant OTUs (BA1 and BA2) were identified (Fig. 6). BLAST analyses suggested that these OTUs could represent a novel species in this genus (Fig. 6). The representative sequence of OTU BA13 showed only 86% identity to *Chitinophaga niabensis* as the closest known species. Phylogenetic analyses of clones in OTU BA13 showed that this culturable OTU is distantly related to known *Chitinophagaceae* bacteria, suggesting that this OTU may represent a novel genus or family in the order *Sphingobacteriales* (Fig. 6 and Supplemental Fig. S2).

### Principal coordinates analyses of clone libraries and isolate collections

**Fig. 7:**
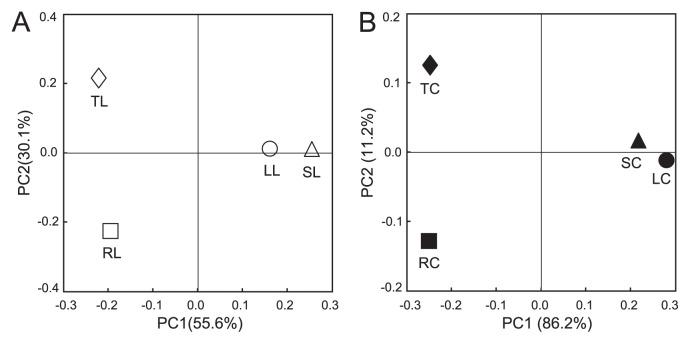
Principal-coordinates analysis of the 16S rRNA gene sequences of clone libraries and isolate collections for potato-associated bacteria derived from field-grown potato plants. The ordinations were constructed for clone libraries (A) and isolate collections (B) using UniFrac distances weighted by the relative abundance. LL, leaf clone library; SL, stem clone library; RL, root clone library; TL, tuber clone library; LC, leaf isolate collection; SC, stem isolate collection; RC, root isolate collection; TC, tuber isolate collection.

The results of PCoA revealed that the community structures of potato-associated bacteria were mainly grouped into above- and below-ground tissues, as supported by PC1 for both clone libraries and isolate collections (Fig. 7A and B). The results also showed that the difference in community structures between root- and tuber-associated bacteria was considerably larger than that between leaf- and stem-associated bacteria.

## Discussion

It has long been known that bacteria naturally inhabit healthy plant tissues of potato plants (10, 17); however, comprehensive assessment of the bacterial diversity of a potato phytosphere has not been studied. In the present study, we conducted bacterial community analyses for a phytosphere of potato plants grown under field conditions by employing both culture-independent and -dependent methods. In the initial attempts, culture-independent analyses without bacterial cell enrichment failed for all potato tissues, because the chloroplast DNA out-competed the bacterial DNAs in the amplification of 16S rRNA genes as template DNA (data not shown). Therefore, the employment of bacterial cell enrichment was thought to be essential for culture-independent assessment of a bacterial community closely associated with potato plants. In general, the diversity observed in culture-independent analysis of an environmental sample is higher than that in culture-dependent analysis, as expected for leaves, stems, and roots in the present study. However, in the case of tuber-associated bacteria in the present study, higher diversity was observed for the isolate collection than for the corresponding clone library (Table 1). Another unexpected result was the extremely low diversity of certain bacterial groups, such as *Betaproteobacteria* and *Bacteroidetes*, in the clone libraries compared to those in isolate collections (Table 2). The low abundance of *Betaproteobacteria* may be attributed to a technical bias caused by the cell enrichment method based on Nycodenz density gradient centrifugation employed in the present study, since Nycodenz density gradient centrifugation is known to recover fewer betaproteobacteria and actinobacteria from soils relative to alpha- and gammaproteobacteria (18). These findings suggest the presence of potential biases in culture-independent analyses, which need to be improved in a future study. Despite these technical problems, the results of community analyses of both clone libraries and isolate collections indicated that the diversity of stem-associated bacteria is extremely low, even in comparison with leaf-associated bacteria (Table 2). In general, a leaf tissue is considered to be a harsher environment as a microbial habitat than a stem tissue, and the diversity of leaf-associated bacteria is often shown to be lower than that of stem-associated bacteria (19, 22). The low diversity of stem-associated bacteria may be one of the characteristics of a potato phytosphere.

The analyses of phylogenetic compositions for both clone libraries and isolate collections revealed that potato-associated bacterial communities are dominated by only a few phyla, mainly consisting of *Proteobacteria*, *Actinobacteria*, *Frimicutes*, *Planctomyces* and *Bacteroidetes* (Table 2). The overall phylogenetic composition at phylum level was consistent with a series of previous studies (4, 9, 25, 34, 42, 49, 50, 52, 53). The *Alphaproteobacteria* and *Actinobacteria* appeared to be dominant bacterial groups in both clone libraries and isolate collections for all tissues.

The detailed phylogenetic analyses identified six dominant genera in *Alphaproteobacteria* (*Rhizobium*/*Agrobacterium*, *Methylobacterium*, *Mesorhizobium*, *Phyllobacterium*, *Caulobacter and Sphingomonas*). Among them, *Rhizobium*/*Agrobacterium* and *Sphingomonas* were observed in all tissues at genus level (Table 2). Two dominant OTUs (AP46 and AP48) showing high similarity to *Agrobacterium larrymoorei* and *Agrobacterium tumefaciens*, respectively, were identified in all potato tissues examined. The pathogenicity and presence of pathogenic genes were examined in isolates belonging to these OTUs by an inoculation test using a tomato seedling and a PCR amplification test. Both examinations were negative for all isolates (data not shown). The genus *Rhizobium*/*Agrobacterium* has been ubiquitously detected in a phytosphere of diverse plant species (5, 22), including potato (11, 42, 52, 53, 57). Meanwhile, dominant OTUs belonging to the genus *Sphingomonas* showed biased distribution to above-ground tissues (OTUs AP1 and AP5) or below-ground tissues (AP6, AP12 and AP13) (Fig. 1), suggesting genetic differentiation at intra-genus level for adapting microenvironments within a phytosphere, as reported for *Pseudomonas* sp. (4). Indeed, the representative sequences of OTUs (AP1, AP2, AP3, AP4, and AP5) for *Sphingomonas* sp. showed high identity to *Sphingomonas faeni* or *Sphingomonas melonis*, both of which have been reported for the association with above-ground tissues of plants (43, 59). In addition, interestingly, an isolate in OTU AP6 showed plant growth-promoting activity to potato seedlings (data not shown). As expected, the genus *Methylobacterium* was exclusively found in above-ground tissues (Table 2), and two dominant OTUs (AP24 and AP26) were found in both clone libraries and isolate collections (Fig. 1). *Methylobacterium* sp. are well known plant-associated bacteria (8), and an isolate of *Methylobacterium* sp. from a potato endosphere has been reported to have antagonistic activity against *Verticillium dahiae* and *Rhizoctonia solani*, two important soilborne pathogens for potato (49). In contrast, two genera in *Phyllobacteriaceae* (*Mesorhizobium* and *Phyllobacterium*) were only found in roots and tubers (Table 2 and Fig. 1). The genus *Caulobacter* was found only in the isolate collections of below-ground tissues (Table 2). Rasche *et al.* reported the dominancy of *Caulobacter* sp. in the endophytic bacterial community by isolating bacteria from lower parts of stems using R2A medium (42).

In the present study, *Betaproteobacteria* were exclusively detected in the isolate collections for below-ground tissues (Table 2). A similar result has been reported by Berg *et al.* (4). Among the *Betaproteobacteria*, *Polaromonas* sp. was shown to be the most dominant genus in both roots and tubers, and three genera, *Variovorax*, *Methylibium* and *Leptothrix*, were mainly detected in roots as predominant groups. The high abundance of the family *Comamonadaceae*, including two genera, *Polaromonas* and *Variovorax*, in a potato rhizosphere has been reported by Sessitsch *et al.* (49). Recently, these genera were considered to be important groups for geochemical cycles of sulfur through desulfonation of aromatic sulfonates in a rhizosphere (47), and could be important for plant nutrition uptake, as freely available sulfur can be limited in arable soils (28, 48). The association of *Methylibium* sp. with potato roots has also been reported in a recent report (34), and an isolate of this genus in the present study showed plant growth-promoting activity in potato seedlings (data not shown). Although the presence of *Leptothrix* sp. in a potato phytosphere has not been reported, interestingly, this species is known for the microbial oxidation of metals such as Fe and Mn, mainly in a rhizosphere of wetland plants (36).

The *Gammaproteobacteria* were shown to be exclusively detected in leaf and stem clone libraries; however, each tissue harbors a totally different phylogenetic composition at lower taxonomic levels. Thus, the genera *Pseudomonas*, *Pantoea* and *Erwinia* were mainly found in leaves (Fig. 2). Two OTUs for the genus *Pantoea* were shown to have high identity to *Pantoea agglomerans*, which has been reported to have antagonistic activity against *Erwinia carotovora* var. *atroseptica*, a pathogen of soft rot (55), and *Pantoea* sp. has been shown to have high persistency in potato stems (49). Meanwhile, the representative sequence of OTU GP3 was identical to *Erwinia chrysanthemi* (Fig. 2), suggesting that healthy potato leaves may harbor a potential pathogen for potato Blackleg. In contrast to leaves, the genus *Acinetobacter* dominated in stems (Table 2). The high abundance of *Acinetobacter* sp. in a potato phytosphere has also been reported in a series of previous studies (3, 43, 52). These studies demonstrated that *Acinetobacter* spp. are highly capable of colonizing in potato plants and are known to function as plant-beneficial microbes (16, 52, 54).

After the *Proteobacteria*, *Actinobacteria* were stably detected at phylum level in both clone libraries and isolate collections for all tissues (Table 2). The genus *Arthrobacter* was relatively abundant in both leaf and stem clone libraries. The corresponding dominant OTU showed high identity to *Arthrobacter ilicis*, which is a pathogen of American holly (Fig. 3). *Arthrobacter* sp. has been detected as an endophyte of potato in several previous reports (13, 49, 57), and an isolate of *Arthrobacter* sp. has been reported to have high activity to promote potato growth (49). Meanwhile, the genus *Microbacterium* dominated in leaf and stem isolate collections (36.1% and 37.7%, respectively). The corresponding OTU AC11 showed high identity to *M. testaceum*. The associations of *M. testaceum* with potato leaves and stems have been reported (3, 42, 49, 66). Becker *et al.* (3) reported that *Microbacterium* sp. was abundantly isolated from a potato phyllosphere regardless of the types of media used. Plant growth promotion has also been reported for *M. testaceum* in potato (49). In contrast, the genus *Streptomyces* was mainly detected for below-ground tissues in both clone libraries and isolate collections. *Streptomyces* sp. can be a source of antagonists of soil-borne pathogens (67). An isolate of OTU AC1 showed growth-promoting activity for potato seedlings (data not shown). Although a causal agent for common scab disease belongs to the genus *Streptomyces*, no OTU closely related to pathogenic spremptomycetes was detected in the present study.

*Firmicutes* was mainly detected in the clone libraries, except for stems. Two genera, *Paenibacillus* and *Bacillus*, were exclusively detected in the clone libraries of below-ground tissues (Table 2). *Paenibacillus* sp. is also known to have antagonistic activity against several pathogens of potato (49). *Bacillus* sp. was exclusively detected in the tuber clone library (Table 2). The corresponding OTU FC13 was closely related to *B. halmapalus* (Fig. 4). Recently, *B. halmapalus* has become known as a source of alpha-amylase for industrial purposes (33). Berg *et al.* (4) reported that two species of *Bacillus* (*B. pumilus* and *B. subtilis*) were isolated throughout a potato phytosphere (4); however, these species were not dominant groups in the present study. Weinert *et al.* (67) have reported that the high abundance of *Bacillus* sp. in the cultural bacterial community of the tuber surface, and showed that the proportion of *Bacillus* sp. on the tuber surface was higher than in the rhizosphere soil. These results suggest the high affinity of *Bacillus* sp. with tubers.

*Bacteroidetes* was mainly detected in isolate collections for below-ground tissues (Fig. 6). In the root isolate collection, the *Bacteroidetes* community was composed of diverse genera with low abundance (Table 2 and Fig. 6). In the tuber isolate collection, half of the isolates of *Bacteroidetes* belonged to the genus *Pedobacter*. Sturz *et al.* (58) reported this genus as a community member of a potato rhizosphere. Recently, Manter *et al.* (34) have identified *Pedobacter* sp. as one of the ten most common genera in root endophytes of potato. It has been reported that an isolate of *Pedobacter* sp. derived from a potato rhizosphere was antagonistic to *Rhizoctonia solani*, a soil-borne pathogen of potato (63).

The results of PCoA for both clone libraries and isolate collections showed distinct and large differences of bacterial community structures between above- and below-ground tissues (Fig. 7). The results also indicated high similarity between leaf and stem communities compared with between root and tuber communities. These results indicate that the tubers harbor a unique community structure which differs from both roots and stems, regardless of the physical or anatomical relationships of these tissues with tubers.

Previous studies of culture-based community analyses showed high similarity between endosphere and rhizosphere communities, and it has been speculated that the majority of endophytes would be derived from the rhizosphere (4, 35, 50, 56); however, in the present study, most dominant taxa at genus or species level showed biased distribution to different tissues, except two OTUs in the *Rhizobium*/*Agrobacterium* group (AP46 and AP48 in Fig. 1). Another interesting difference between the present and previous studies was the abundance of *Pseudomonas* species. *Pseudomonas* sp. has been reported as one of the most dominant genera throughout all tissues of the potato phytosphere in previous studies (14, 49, 52, 53, 57). In contrast to these studies, this bacterial group was only predominant in the leaf clone library in the present study (Table 2).

Recently, community analyses of the potato rhizosphere have been conducted with pyrosequencing by two groups. Manter *et al.* (34) reported 238 known genera in 15 phyla and found 477 OTUs with 97% identity, as for root endophytes. Inceoğlu *et al.* (24) reported 450 genera in 25 phyla of the bacterial community of a rhizosphere soil, while we identified 82 genera from 8 phyla and found 237 OTUs across an entire phytosphere by one-pass sequencing. Despite the differences in the sample preparations and the methodologies employed, all of these studies showed that a potato-associated bacterial community is composed of a few highly dominant taxa with numerous rare species. Similar results have been observed in our previous community analyses of above-ground tissues of soybeans (19, 22); therefore, such community structures could be one of the features of plant-associated bacteria.

In conclusion, in the present study, the community structures of potato-associated bacteria in both above- and below-ground tissues were comprehensively examined by analyzing clone libraries and isolate collections. The results indicated that each microenvironment in a potato phytosphere harbors a distinct community structure. The results also suggested that genetic differentiation at intra-genus level is present for most potato-associated bacteria to adapt to microenvironments within a potato phytosphere. In addition, it is well known fact that culture-dependent and -independent analyses often show considerable differences in taxonomic composition due to the unavoidable biases present in both analyses, as observed in previous studies as well as in the present study (3, 6, 41, 62). At this moment, the employment of both culture-dependent and -independent methods seems to be recommended for comprehensive analyses of the diversity of a phytosphere community. As shown in the present study, comprehensive analyses of plant-associated microbes would provide basic ecological information and would lead to knowledge-based utilization of beneficial microbes in an agronomic environment.

## Supplementary Material



## Figures and Tables

**Table 1:** Characteristics of clone libraries and isolate collections derived from potato tissues

	Clone libraries				Isolate collections			
		
	Leaf	Stem	Root	Tuber	Leaf	Stem	Root	Tuber
Statistics								
No. of sequences	150	174	169	161	205	175	198	212
No. of OTUs (97% identity)^a^	40	26	101	46	9	16	57	54
No. of singletons	18	9	75	26	3	5	25	27
Library coverage (%)^b^	88.0	94.8	55.6	83.9	98.5	97.1	87.4	87.3
Diversity indexes								
Chao1	70.6	36.2	327.1	86.6	12.0	18.0	84.3	83.3
ACE	73.2	51.1	695.7	134.8	28.1	19.7	106.6	168.1
Shannon index (*H*′)	3.3	2.4	4.3	3.0	1.6	1.9	3.6	3.3
Simpson index (1/*D*)	23.1	7.3	81.1	11.9	4.2	4.6	28.7	19.7

a OTUs were defined at 97% sequence identity.

b *C_X_*=(*n*/*N*), where *n_x_* is the number of singletons that are encountered only once in a library and *N* is the total number of clones.

**Table 2:** Phylogenetic compositions of 16S rRNA gene libraries and isolate collections derived from potato tissues

Phylogenetic compositions (%)^a^	Clone libraries				Isolate collections			
	
	Leaf	Stem	Root	Tuber	Leaf	Stem	Root	Tuber
*Proteobacteria*	84.0	89.7	65.7	62.0	62.4	57.7	72.9	60.4
*Alphaproteobacteria*	40.7	43.7	32.5	50.0	62.4	53.7	38.2	42.5
*Methylobacterium*	9.3	7.5	—	1.9	41.0	35.4	—	—
*Rhizobium/Agrobacterium*	18.7	29.9	8.9	18.0	2.4	—	6.0	8.5
*Mesorhizobium*	—	—	4.7	3.7	—	—	5.5	3.3
*Phyllobacterium*	—	—	3.0	14.3	—	—	1.5	1.4
*Caulobacter*	—	—	—	—	—	—	8.5	14.6
*Devosia*	0.7	—	3.6	—	—	—	2.0	0.9
*Sphingomonas*	8.0	3.4	1.8	8.1	19.0	17.7	8.0	2.4
Other genera	4.0	2.9	8.1	3.4	—	0.6	4.2	4.8
Unclassified								
*Alphaproteobacteria*	—	—	2.4	0.6	—	—	2.5	6.6
*Betaproteobacteria*	3.3	—	5.9	5	—	4.0	31.7	17.5
*Polaromonas*	—	—	—	3.7	—	—	8.5	8.5
*Variovorax*	—	—	—		—	2.3	3.5	0.5
*Pelomonas*	—	—	—	—	—	—	2.5	4.7
*Methylibium*	—	—	—	—	—	—	4.5	—
Other genera	3.3	—	5.9	1.3	—	1.7	12.7	3.8
*Gammaproteobacteria*	40.0	46.0	26.6	5.0	—	—	3.0	0.5
*Acinetobacter*	—	36.2	—	—	—	—	—	—
*Pseudomonas*	10.0	0.6	0.6	2.5	—	—	—	—
*Erwinia*	4.7	1.1	—	—	—	—	—	—
*Pantoea*	6.0	2.9	—	—	—	—	—	—
Other genera	3.3	1.8	5.2	1.9	—	—	3.0	0.5
Unclassified								
*Enterobacteriaceae*	16.0	3.4	—	—	—	—	—	—
Unclassified								
*Chromatiales*	—	—	3.6	—	—	—	—	—
Unclassified								
*Gammaproteobacteria*	—	—	17.2	0.6	—	—	—	—
*Deltaproteobacteria*	—	—	0.6	—	—	—	—	—
*Actinobacteria*	9.3	10.3	10.7	5	37.6	39.4	14.6	17.0
*Microbacterium*	—	1.1	—	—	36.1	37.7	—	1.4
*Arthrobacter*	6.7	8.0	1.2	—	—	0.6	0.5	—
*Streptomyces*	—	—	4.1	1.2	—	—	6.5	2.8
Other genera	2.6	1.2	5.4	3.8	1.5	1.1	7.6	12.8
*Firmicutes*	6.7	—	10.1	32.0	—	0.6	0.5	0.5
*Paenibacillus*	—	—	7.1	5.0	—	—	—	—
*Bacillus*	1.3	—	3.0	25.0	—	0.6	—	0.5
Other genera	5.4	—	—	2.0	—	—	0.5	—
*Bacteroidetes*	—	—	2.4	—	—	2.3	12.1	21.7
*Pedobacter*	—	—	—	—	—	2.3	1.0	12.3
*Chitinophaga*	—	—	0.6	—	—	—	3.5	0.5
*Lacibacter*	—	—	—	—	—	—	—	4.2
Other genera	—	—	1.8	—	—	—	7.6	4.7
*Planctomycetes*	—	—	8.9	—	—	—	—	—
*Schlesneria*	—	—	4.1	—	—	—	—	—
Other genera	—	—	4.8	—	—	—	—	—
*Verrucomicrobia*	—	—	1.2	—	—	—	—	—
*Acidobacteria*	—	—	0.6	—	—	—	—	—
Bacteria_incertae_sedis	—	—	0.6	—	—	—	—	—
Unclassified Bacteria	—	—	—	1.9	—	—	—	0.5

a 16S rRNA gene sequences were classified by RDP Classifier. The compositions of genera are shown for only dominant groups.
