# Supplementary material for: Molecular Characterization of the Bacterial Community in a Potato Phytosphere
Source: Microbes Environ. 2013 Jun 8;28(3):295–305. doi: 10.1264/jsme2.ME13006 (PMC4070957; doi:10.1264/jsme2.ME13006)
Supplement: Supplementary file 1 [file 28_295_s1.pdf]

Supplemental Material

Supplementary Figure Legends

Fig. S1. Phylogenetic distribution of OTUs of *Planctomycetes* based on the 16S rRNA gene sequences of the clone libraries and isolate collections derived from field-grown potato plants. The dendrogram indicates the phylogenetic relationships among the representative sequences of OTUs (defined by ≥97% identity). The table indicates the relative abundance of clones or isolates belonging to each OTU in each library or collection and the results of a BLAST search using the representative sequences. Shading indicates OTUs described in the main text.

Fig. S2. Phylogenetic tree analysis of a potato tuber-associated *Chitinophagaceae* bacterium. The tree was constructed by the neighbor-joining method with the reference sequences in the family of *Chitinophagaceae* and the representative sequence of OTU BA13 derived from the tuber clone libraries of 16S rRNA genes. The scale represents 0.1 substitutions per site. The numbers at the nodes are the proportions of 1,000 bootstrap re-samplings, and values <50% are not shown. *Flavobacterium aquatile* was used as an outgroup.

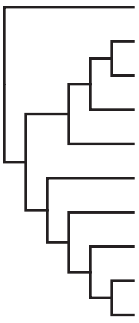

	Clone libraries				Isolate collections						Identity
OTUs	Leaf	Stem	Root	Tuber	Leaf	Stem	Root	Tuber	Closest known species	Acc. No.	(%)
PL1	-	-	0.6	-	-	-	-	-	*Rhodopirellula baltica*	HQ845505	83
PL2	-	-	0.6	-	-	-	-	-	*Cymbella ventricosa*	HQ703548	88
PL3	-	-	0.6	-	-	-	-	-	*Cymbella ventricosa*	HQ703548	93
PL4	-	-	0.6	-	-	-	-	-	*Gemmata obscuriglobus*	X81957	90
PL5	-	-	0.6	-	-	-	-	-	*Gemmata obscuriglobus*	X81957	96
PL6	-	-	0.6	-	-	-	-	-	*Planctomyces limnophilus*	CP001744	88
PL7	-	-	1.2	-	-	-	-	-	*Planctomyces limnophilus*	CP001744	89
PL8	-	-	0.6	-	-	-	-	-	*Schlesneria paludicola*	AM162407	98
PL9	-	-	0.6	-	-	-	-	-	*Schlesneria paludicola*	AM162407	90
PL10	-	-	3.0	-	-	-	-	-	*Schlesneria paludicola*	AM162407	93

Fig. S1

Someya et al., 2013

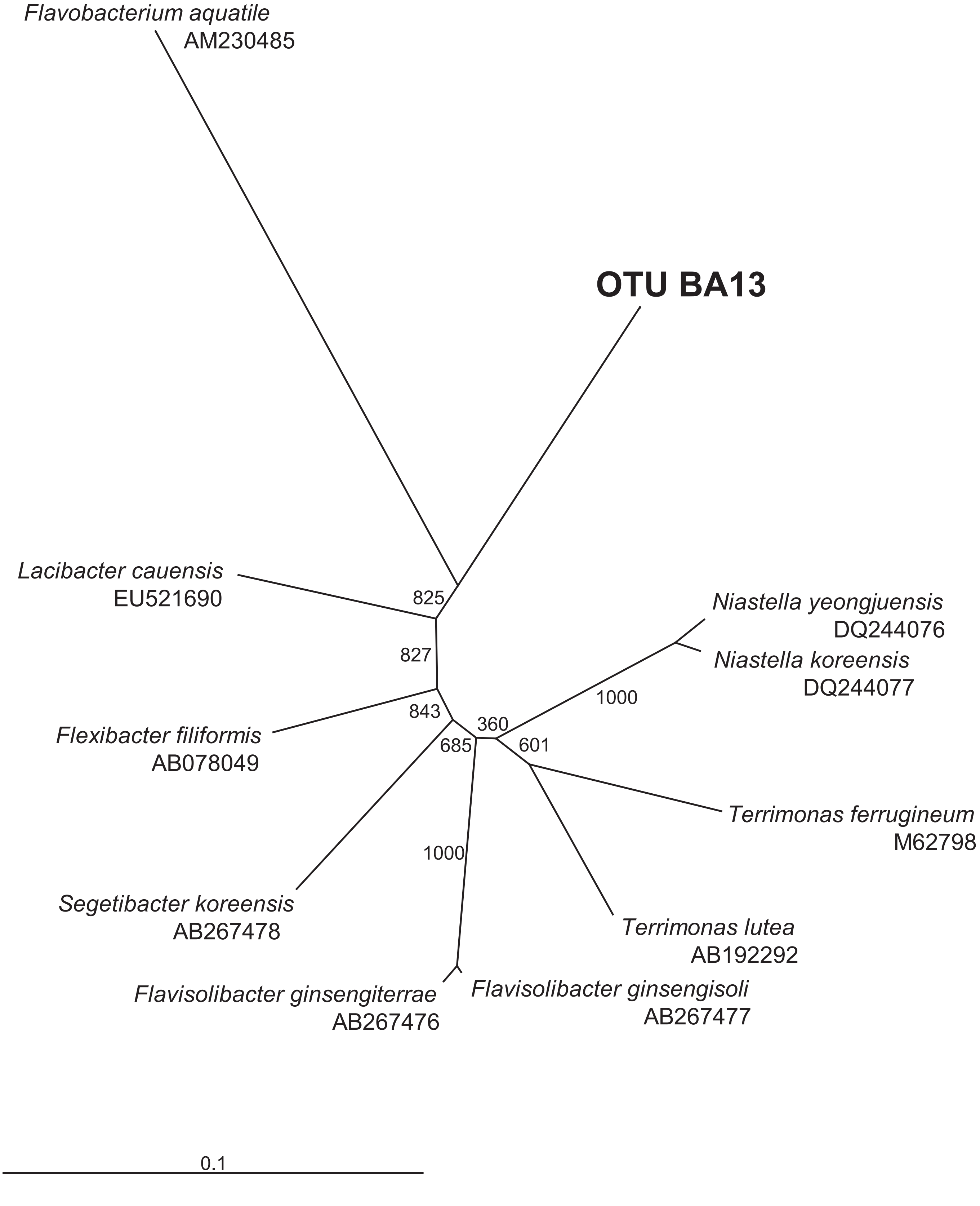

Fig. S2

Someya et al., 2013
